# Kaumātua Mana Motuhake: A study protocol for a peer education intervention to help Māori elders work through later-stage life transitions

**DOI:** 10.1186/s12877-019-1041-2

**Published:** 2019-02-07

**Authors:** John G. Oetzel, Brendan Hokowhitu, Mary Simpson, Rangimahora Reddy, Sophie Nock, Hineitimoana Greensill, Michael P. Cameron, Pare Meha, Kirsten Johnston, Truely Harding, Pita Shelford, Linda Tuhiwai Smith

**Affiliations:** 1 University of Waikato, Private Bag 3105, Hamilton, 3240 New Zealand; 2 Rauawaawa Kaumātua Charitable Trust, 50 Colombo St, Hamilton, 3204 New Zealand

**Keywords:** Tuakana-teina, Peer education, Positive ageing, Community-based participatory research, Mana motuhake

## Abstract

**Background:**

The Aotearoa/New Zealand population is ageing and numerous studies demonstrate with this phenomenon comes increases in non-communicable diseases, injuries and healthcare costs among other issues. Further, significant inequities exist between Māori (Indigenous peoples of Aotearoa/New Zealand) and non-Māori around poor ageing and health. Most research addressing these issues is deficit oriented; however, the current research project takes a strengths-based approach that highlights the potential of kaumātua (elders) by asserting mana motuhake (autonomy, identity and self-actualisation). We believe that the esteem of elders in Māori culture signals transformative potential. Specifically, this project utilises a ‘tuakana-teina’ (older sibling/younger sibling) peer-educator model, where kaumātua work with other kaumātua in relation to health and wellbeing. The objectives of the project are (a) to develop the capacity of kaumātua as peer educators, whilst having positive impacts on their sense of purpose, health and wellbeing; and (b) to enhance the social and health outcomes for kaumātua receiving the intervention.

**Methods:**

The research is grounded in principles of Kaupapa Māori and community-based participatory research, and brings together an Indigenous community of kaumātua, community health researchers, and academic researchers working with two advisory boards. The project intervention involves an orientation programme for tuakana peer educators for other kaumātua (teina). The research design is a pre- and post-test, clustered staggered design. All participants will complete a baseline assessment of health and wellbeing consistent with Māori worldviews (i.e., holistic model). The tuakana and teina participants will be divided into two groups with the first group completing the intervention during the first half of the project and the second group during the second half of the project. All participants will complete post-test assessments following both interventions allowing comparison of the two groups along with repeated measures over time.

**Discussion:**

The findings will provide an evidence base for the importance and relevancy of kaumātua knowledge to create contextually based and culturally safe age-friendly environments that facilitate engagement and participation by kaumātua for kaumātua. If the model is effective, we will seek to facilitate the dissemination and scalability of the intervention.

**Trial registration:**

Australia New Zealand Clinical Trial Registry (ACTRN12617001396314); Date Registered: 3 October 2017 (retrospectively registered).

**Electronic supplementary material:**

The online version of this article (10.1186/s12877-019-1041-2) contains supplementary material, which is available to authorized users.

## Background

The Aotearoa/New Zealand (hereafter referred to as ‘Aotearoa’) population is ageing and numerous studies demonstrate with this advancing age comes increases in non-communicable diseases, injuries and healthcare costs among other issues [1]. Accordingly, there is a general assumption that the increasing percentage of older people over the first half of the twenty-first century will lead to an increasing burden on the socio-economic system [1]. Further, significant inequities exist between Māori (Indigenous people of Aotearoa) and non-Māori around poor ageing and health outcomes [2–4], that, in turn implicate individual, economic, social and cultural costs [2–4]. This assumption has been central to public discourses surrounding Aotearoa’s ageing population and is underpinned by a deficit model emphasising limitations, weakness, and dependency [2]. Certainly ageing is associated with increased disease rates and costs, yet there are also many positive elements of ageing. Thus, the central discourse surrounding the ageing population needs to shift if Aotearoa is to re-frame older people as highly valuable assets to society. The current research project takes a strengths-based approach that highlights the potential of kaumātua (elders). We believe that the esteem of elders in Māori culture in principle (if not always in practice) signals transformative potential. Our approach is part of the Ageing Well National Science Challenge in Aotearoa (https://www.ageingwellchallenge.co.nz/), which looks to provide more focus on positive ageing as part of the government’s strategic approach to science investment.

Consequently, the present research emphasises the strengths-based approach as grounded in an Indigenous philosophy within a culture that is often overlooked in relation to how it can influence New Zealand’s national culture in general and provide innovations in socio-economic spaces. Māori culture venerates its elderly, such is its centrality to a Māori way of life; from the way that typical Māori whānau (extended family) operate beyond the bounds of the traditional western family model to tikanga (customs and protocols) on the marae (community meeting space) and in various community settings [5, 6]. As Sir Hirini Moko Mead states: “The kaumātua and kuia, the elders, are often the guardians of tikanga. They are expected to know” and “Experience is definitely helpful in knowing what to do [6] (p. 14).” Mead also noted that kaumātua have active roles in the community including governance and mediating disputes. Further, kaumātua are often the glue that hold fragmented extended families together through raising grandchildren [7].

Nonetheless, Māori, like all other people, face significant transition points as they age, such as loss of independent living (e.g., loss of driver’s license), loss of a spouse, and changing health conditions [1, 8, 9]. Successfully navigating these transitions depends on being able to manage emotional and socio-economic factors, as well as service systems, whilst often being reliant on family or whānau [10–14]. Kaumātua who are unable to successfully navigate significant transition points during ageing are exposed to many negative consequences including poor quality of life, social isolation, and poor health outcomes [15, 16]. These transitions for kaumātua are substantial and different than in other ageing populations given the context of significant health and social inequities. Thus, these transitions need to be addressed directly to facilitate positive ageing.

### Kaumātua Mana Motuhake

Mana motuhake is a concept that foregrounds independence and autonomy to achieve actualisation—including collective determination and independence. In this manner, kaumātua assert their independence and autonomy so they can live a life of longevity and quality for self and others [17]. Historically, kaumātua have faced a dominant society that has failed to realise their full potential as they age [18]. Yet, for Māori, the elderly are “carriers of culture, anchors for families, models for lifestyle, bridges to the future, guardians of heritage, and role models for younger generations [19] (p. 14).” The current project is invested in upholding tino rangatiratanga (independence and autonomy) and mana (status and prestige as viewed by self and others) via a culturally appropriate *‘*tuakana-teina’ peer-educator model where kaumātua work with other kaumātua in relation to significant life-transitions [17]. Accordingly, this research values older people in all settings and views their experience and status as key tools for positive ageing. Further, this research is grounded in Māori epistemologies surrounding ageing [20] and provides insights into how a Māori worldview of ageing and Māori practices surrounding ageing have the potential to improve life courses for all New Zealanders.

Whilst the research is grounded in a strengths-based approach, it does not assume that the tikanga surrounding Māori elders described above is consistent or practiced by all Māori. Rather the research imbibes cultural concepts into a peer-educator/peer-support model to help shift practices in working with elders to address significant life-transitions and to ideally help change the dominant public discourse surrounding ageing. The research includes Māori epistemology and worldviews in a way that is accessible to all (e.g., including materials in both English and Māori languages). Thus, the ageing population is valued and has mana motuhake—that is, self-determination as their life-situations evolve including the responsibility of taking on new and vital roles.

### Tuakana/Teina peer-educator/peer-support model

The research extends existing understandings related to the improvement of outcomes for kaumātua by focusing on the possibilities of using elders as peer educators via the Indigenous concepts of tuakana-teina. Tuakana-teina is a Māori customary concept [6] signifying the importance of the relationship between an elder and younger sibling, or cousin, of the same sex; however, it can also be based on experience and training and hence why it was chosen for this project. The concept is based on responsibility and reciprocity, and can be pedagogically applied in peer-education mentor/mentee settings [21]. The tuakana-teina peer-education pedagogy in the present research aligns with this central philosophy, where the reciprocal relationship is underpinned by self-efficacy and responsibility. The tuakana role includes culturally resonant values, principles, and practices determined by the spiritual balance between tapu (sacred) and noa (ordinary); the exercise of mana, pono (truth, integrity, faithfulness), aroha (love, compassion, mercy, empathy) and tika (correctness, justice, fairness). Pono concerns the ethics of how tapu is addressed and mana is exercised [6, 22].

With the ultimate goal of promoting a self-determined life and improving health and social outcomes, this peer-education approach essentially encourages self-efficacy and mana for both the tuakana and the teina, whilst profiting social integration and engagement. More practically, both parties are not only able to socially interact with each other, the teina receives valued support that may prevent social isolation as kaumātua work through life-events, whilst the tuakana is given further meaning and engagement via the self-efficacy that supporting another can enable. Inherently, the peer-education model develops age-friendly social environments that value knowledge and cultural concepts such as whakawhanaungatanga (making social connections), tautoko (advocacy, support) and mātauranga (Indigenous knowledge) [5, 6, 21].

Drawing on well-known theories including social learning theory [23], theory of reasoned action [24], and diffusion of innovation theory [25], the research literature demonstrates that peer education/support is an effective method of enhancing a variety of health, social, and economic conditions [26–29]. Peer support and education include a range of assistance activities provided by lay individuals (non-professionals) to others experiencing a health or social need who are members of the same age, health, culture, ethnicity, or other cohort [30]. Peer support is different from everyday whānau and community support networks where people help each other because of existing relationships. It is also different from organisation-driven, “para-professional” support—even though such programmes may use peer supporters. Peer education/support utilises lay individuals and relies on a created social network based on shared health or social service needs. Such networks are commonly created by health professionals and social service agencies but may include self-help groups [30].

Peer education/support is used to help people manage various ‘age and stage’ life-transitions, including acute and chronic situational health and social stressors [30]. Peer educators are mostly used with younger populations, but elder peer educators have recently featured in self-management of chronic conditions [31], raising awareness of health [32, 33], physical activity and fall-prevention in older age [34–37], successful ageing [38], and palliative care [39–41]. In addition to improving health outcomes of recipients, peer education/support has also shown positive outcomes for peer educators’ own sense of wellbeing and maintenance of health self-management and programme engagement [42–44].

### Research aims

We seek to address the mana motuhake of kaumātua, asking: What are the outcomes of a ‘tuakana-teina’ peer-educator model, where kaumātua work with other kaumātua in relation to wellness, social integration/connectedness, engagement, life-enhancement, independence, and, in particular, significant life-transitions? The project has four specific objectives: (a) To develop the capacity of kaumātua as peer educators/supporters and have positive impacts on their sense of purpose and health and wellbeing; (b) To enhance the social and health outcomes for kaumātua receiving the intervention; (c) To determine the cost-effectiveness of the intervention; and (d) To determine whether and how the intervention could be scalable through effective dissemination and implementation processes.

## Methods

### Methodology

The project is guided by Kaupapa Māori [45, 46] and a community-based participatory research approach (CBPR) [47, 48]. Kaupapa Māori and CBPR are collaborative research processes that underpin every phase of the research process. In New Zealand considerable advances have been made in working with mātauranga, science and other disciplines to develop innovative methods that are non-exploitative of Māori communities, that work with their strengths rather than deficits and that lead to advancement of mutually beneficial knowledge and applications. These two approaches emphasise this mission and ensure that the project is kaumātua and end-user led. With their wealth of life experience and mātauranga, kaumātua are strongly placed to extend their existing significant cultural roles to include the spread of knowledge needed to empower other kaumātua and their whānau in life-transitions. The Kaupapa Māori approach here centralises the mana motuhake of kaumātua as having value and at the same time responsibility [49].

In the last 30 years, Kaupapa Māori has emerged as a key discourse theory and praxis [46] and, in the context of the present research, provides a culturally appropriate methodology as it normalises Māori worldviews and practises [50]. Indigenous academics, theorists, activists, and researchers agree that Kaupapa Māori is an approach that focuses on self-determination and local context by prioritizing Indigenous aspirations and history [46, 51]. The key concepts of this approach are the validity of a Māori epistemology; the legitimacy of the rights of Māori as Indigenous peoples [51] and the importance of tikanga and mātauranga [45, 46]. Mātauranga Māori is the knowledge that underpins a Māori epistemology and provides the basis of technological and philosophical skills of the community; it is a by Māori for Māori approach to research [52, 53].

CBPR is a collaborative research approach that equitably involves academic and community partners in the research process and recognises the unique strengths that each partner brings [47]. Following the practice of many CBPR partnerships and Kaupapa Māori principles [46, 47], we use two advisory boards to guide our work: (a) a Board Advisory Group; and (b) an Expert Advisory Group. The Board Advisory Group is comprised of trustees of Rauawaawa Kaumātua Charitable Trust (RKCT; an organisation that serves the health and social wellbeing needs of elders using a Māori philosophy) who are all kaumātua themselves. This was fundamental to the methodology because of the centrality of representatives of the research participants to the decision-making process of the project. It provides oversight, guidance and input into all the research methods, procedures, data-collection processes, and analysis that the research team is conducting in this project. The Expert Advisory Group includes experts in health and social issues related to the project. It ensures the intervention and research design aligns with best practices in ageing transitions. We meet with these groups bi-monthly during the development of the research design and intervention and quarterly through the duration of the project.

Our research team involves an ongoing research partnership between a community organisation (i.e., RKCT), and academics from multiple disciplines from the University of Waikato including communication, public health, economics, and Māori and Indigenous Studies. We underwent training during the first month of the project on cultural safety, research protocols and ethics, CBPR and Kaupapa Māori principles to support our research methodology and ensure all researchers were clear about expectations.

### Intervention development

We will develop a “Tuakana-teina/peer education orientation programme” for life-transitions of kaumātua. The programme is framed as an ‘orientation’ instead of a training programme to reinforce the research project’s participant driven nature, whilst at the same time making it clear that the project itself will draw on the extant knowledge and expertise of kaumātua themselves. This programme development will be informed by knowledge generated from our previous research projects and other related research about culturally relevant protocols and processes during life-transitions for kaumātua. Consultation and interaction with tuakana (peer supporters) will inform the development and delivery of the orientation material. Teina (peers learners) also will have input into the effectiveness of the intervention’s implementation, and areas of strength and weakness in the processes used.

Our own research and RKCT’s service providers identified key challenges, barriers, and opportunities related to the life-transitions as part of the proposal writing. Together these provided a solid foundation for the orientation programme. Although we investigated other research [41, 54, 55], the primary concern will be to develop a Māori model of peer support for use with kaumātua. As part of the proposal writing, we conducted a literature review of critical cultural perspectives and values, including Te Ao Māori (worldview); mātāpono (principles) and tikanga; kaumātua roles; tuakana and teina as roles and concepts; and Māori approaches to communication. In addition, we reviewed peer support as it is discussed in Māori and Indigenous contexts, as well as the ageing, education, and health literature.

The development of the intervention will include extensive consultation with kaumātua, the Board Advisory Group, and Expert Advisory Group to ensure that tikanga and Māori communication approaches remain central. We will keep minutes of all advisory group meetings, meetings of the team that developed the Tuakana Orientation Programme, and consultation sessions with kaumātua. These practices will ensure the integrity of the development process. We will run a full, three-session pilot programme with kaumātua from a different region and organisation. This pilot will inform the final design of the Tuakana Orientation Programme; we will create a manual for the intervention that includes instruction for orientation, implementation and evaluation.

### Orientation Programme process

The orientation of the tuakana will be completed at two time periods for Tuatahi (first group, T1) and Tuarua (second/comparison group, T2); these will correspond to when each group commences the intervention with teina. Orientation activities will be run by members of the research team with support from the Board Advisory Group. The orientation will include sharing of: (a) Māori values and mātāpono; (b) definitions of tuakana-teina/peer support; (c) four kinds of support; (d) forms of Māori communication; and (e) specific communication tools to support the tuakana in their conversations with teina. The final program will be comprised of four 4-h sessions over two weeks. Each session includes breaks for morning tea and lunch. The initial sessions focus on exploration of the programme, with the later sessions focusing on the tuakana-teina relationship, skill development, and communication practice. “Booster sessions” will be offered to follow-up on the information and to provide additional opportunities to practice the skills.

### Study design

**Fig. 1:**
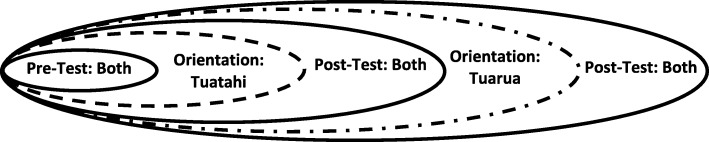
Study Design

The research design for the evaluation of the orientation programme is a pre- and post-test, clustered staggered design with T1 (target *n* = 15) and T2 (target *n* = 15) groups. T1 will participate in the orientation programme initially, while T2 will participate in a subsequent orientation. The peer educator capacity of tuakana will be assessed at three stages: pre-intervention, post-intervention for the T1 group, and post-intervention of the T2 group. After the orientation programme, each tuakana will talk with each teina at least three times to address the specific life-transitions of teina. Teina also will complete three evaluations with variations for the two groups: (a) T1: pre-intervention, approximately four months post-intervention, and then four months after the second evaluation; (b) T2: pre-intervention, approximately four months after initial survey, and then four months post-intervention. This staggered approach was approved by the Board Advisory Group during the original proposal as fitting the mission of the organisation and the needs of kaumātua. Consistent with Kaupapa Māori principles, withholding an intervention from participants is not ethical. Thus, the research design enables a rigorous comparison of the orientation programme whilst ensuring that all teina receive the intervention. Further, the use of a staggered design is a strong, and pragmatic design for interventions in the health service sector [56]. Figure 1 illustrates the staggered research design. The project is registered with the Australia New Zealand Clinical Trial Registry (ACTRN12617001396314).

### Sample and recruitment

Tuakana will be purposively sampled to include kaumātua with effective peer educator attributes. The advisory groups considered the relevant peer educator attributes for ageing research [41] and attributes within Te Ao Māori such as mana, pono, aroha and tika [6, 19]. Tuakana will receive koha (an offering) towards their participation in the programme, conversations with teina, and data collection over the duration of the project (approximately $45/event).

The project will have a target of 180 teina participants randomly selected from the RKCT client base of approximately 600 kaumātua that affiliate to various iwi (tribes). Tuakana will be removed from the sampling frame. Teina will be assigned to a tuakana with matched biological sex. The only exclusion criteria is ethnicity (i.e., not being Māori). Decline and retention rates (including reasons such as withdrawal and passing away) will be tracked. Teina will receive koha as a contribution towards their participation in conversations and data collection (approximately $40/event). Tuakana and teina will be assigned to T1 and T2 groups ideally using random assignment although convenience assignment will be accommodated and tracked (e.g., when they were able to participate or to match tuakana/teina).

The sample sizes of tuakana and teina were determined by a balance of resources and statistical power to determine the optimal design. We conducted power and sample size estimates based on the research design [57] and confirmed with the ‘od.exe’ software package [58]. The following assumptions were made: (a) probability value = .05; and (b) rho (intra-class correlation) = .10. Six teina for each tuakana results in power of .80 with a medium effect size (d = .55). A medium effect size was chosen given prior research on the impact of elder peer educators on self-rated health and related wellbeing outcomes [37, 38].

### Measures

The measures included in this study centre on two core constructs: hauora (health) and mana motuhake (see Table 1 for a summary). Hauora is conceptualised using the whare tapa wha (four walls of a meeting house) model of health and wellbeing developed for Māori communities [59]. The model includes tinana (physical/body), hinengaro (mind/thought/intellect), whanaungatanga (social) and wairua (spiritual). Hauora is a necessary (although not sufficient) condition for achieving mana motuhake. Measures of these two supra-constructs include both quantitative and qualitative questions and will be part of both tuakana and teina surveys across the three-time periods. We will include demographic questions only at pre-intervention (e.g., ethnicity, age, relationship status, care of grandchildren, and who else lives with them). We also will include additional measures related to the intervention. The total number of questions were limited and short measures were used in awareness of response burden for kaumātaua.

**Table 1:** Constructs and Measures

Construct	Measures	Number of Items
*Pre- and Post-Test Measures*		
Hauora—tinana/hinengaro	Self-rated health	1
Hauora—tinana/hinengaro	Health related quality of life—physical and mental wellbeing	7
Hauora-hinengaro	Knowledge and likelihood of using services	3
Hauora-wairua	Spirituality	1
Hauora-whanaungatanga	Loneliness	4
Hauora-whanaungatanga	Cultural connection	4
Hauora-whanaungatanga	Social support	4
Hauora-whanaungatanga	Perceived burden & benefit	4
Mana motuhake	Economic wellbeing	3
Mana motuhake	Perceived autonomy	3
Mana motuhake	Global life satisfaction	1
Demographics	Living situation	3
*Intervention Measures*		
Mana motuhake	Satisfaction with interaction	3
Mana motuhake	Sense of purpose	3
Mana motuhake	Self-efficacy as a tuakana	5
Mana motuhake	Perceived effectiveness of orientation programme	4

### Hauora

For physical and mental health, we will include a single item for self-rated health [60, 61] and seven items adapted from the Medical Outcomes Study [62] as measures of health-related quality of life (HRQOL). These are well-established and valid approaches for measuring HRQOL although the adaption will result in a need to re-establish factorial validity. Also in reference to physical/mental health, we developed new questions related to knowledge of health and social services. These are primarily open-ended questions to explore participants’ understanding of health services related to life transitions. An additional file lists all questions created specifically for this study in English and Māori (see Additional file 1). For spirituality, we will include a single item that came from Te Kupenga (the Māori Social Survey run by Statistics New Zealand) focused on strength of spirituality [63].

For social health and wellbeing, we will include four different measures. Loneliness will be measured by three items from a study of older New Zealanders [1] and one item created for this study. Cultural connection to iwi and hapū (subtribe) will be measured by four items from a study of older Māori [16]. Social support will be measured by four items from a scale used in the study of older New Zealanders although the scaling was changed from dichotomous to ordinal [64]. Finally, perceived burden and benefit on the family will be measured by four items; two items on burden came from a study of self-perceived burden [65] and the two benefit items were created for this study. See Additional file 1 for items created for this study for the tuakana and teina questionnaires; see Additional file 2 for items created for this study for tuakana questionnaires only.

### Mana Motuhake

We will include three quantitative measures of mana motuhake and several open-ended questions for both tuakana and teina. Economic wellbeing focuses on needs related to paying bills, quality of housing, and control of resources. Two items come from Te Kupenga and one was created for this study [63]. Perceived autonomy examines the independence that kaumātaua self-reported and will be measured with two items from a previous study [66] and one item from Te Kupenga. Global life satisfaction will be measured using the Cantril ladder [67]. Finally, we will ask five open-ended questions about general life concerns, concerns in the next few years, best things in life, what would improve their lives and how they assess their own mana motuhake. For tuakana only, we also will ask questions about sense of purpose. Three self-reported items will be included based on a previous study [68].

### Intervention-related measures

Several measures of mana motuhake will be directly related to the intervention. Based on a guideline for creating self-efficacy scales, five items were created for this study to assess the perceived self-efficacy of tuakana in relation to their ability to fulfil the role of tuakana [69]. Using three items created for this study and one item from a previous study [70], the perceived effectiveness of the orientation programme will be determined by the tuakana. For both tuakana and teina, we will include three items created for this study about the perceived satisfaction of the interaction with each other in terms of the time spent, interaction, and advice provided.

Additional measures about the intervention will relate to the conversations between tuakana and teina. Tuakana will have at least three conversations with each teina over a two-three month period. Checklists will be provided to the tuakana to help structure their conversations with teina around specific aspects to be covered in the orientation programme. The conversations will be audio recorded with the permission of the participants. A research team member will code the conversations to check the fidelity of the conversations to the orientation programme. The coding sheet will be a similar checklist given to the tuakana. Coders will be trained by one of the senior research team members on the coding procedures and rules. A separate researcher will code approximately 10% of the interactions to establish inter-coder reliability prior to completion of the primary coding.

The intervention will enable participants to talk about their transitions and enhance meaning about them. There is a chance that T1 participants will talk with T2 participants in the other group; this is understandable and not something we wish to control. We will measure whether participants talk to other kaumātua at RKCT about the project/life transitions and used these measures as confounders in the analyses. These measures mitigate impact on research design.

### Cultural safety and data collection procedures

We are mindful that ageing and life-transitions are sensitive topics and, thus, utilised our prior experience to protect kaumātua participant confidentiality and provide cultural safety. To this end, we developed a detailed health and safety plan at the beginning of the project, working with both advisory groups, for the intervention orientation and data collection to ensure cultural safety for participants. The research procedures were approved by the University of Waikato Human Research Ethics Committee through the Faculty of Māori and Indigenous Studies.

The participants will be able to complete the questions on their own or via a structured interview and have a support person present if they desire. The questionnaire includes both English and Māori language versions. The Māori language version was developed through a translation and back-translation procedure to establish equivalence. Finally, the questionnaire is written with large font and with plenty of spacing between questions to support kaumātua in reading the content.

### Data analysis plan

#### Pre-test data

Using the pre-test data, we will (re-)establish the psychometric properties of measures. Factorial and discriminant validity will be assessed with confirmatory factor analysis (CFA) and reliability established with Cronbach’s alpha. Fit indices will include χ^2^/degrees of freedom (χ^2^/df), comparative fit index (CFI), Tucker-Lewis index (TLI), and standardized root mean square residual (SRMR). Good fit was determined if CFI and TLI were at or above .90 with .95 as ideal, SRMR was less than .08 with .06 as ideal, and χ^2^/df < 3 or less than 2 as ideal [71]. Analysis will be completed with AMOS 25.0. We will confirm specific measures and also consider high-order factor models of mana motuhake and hauora. Convergent validity will be established using Pearson correlations among the measures (using SPSS 25.0).

Multivariate models of several dependent variables (i.e., HRQOL, perceived autonomy, loneliness, and sense of purpose) will be constructed using hierarchical regression. Variables will be entered in two blocks: demographics and other variables. We will simplify the models by removing variables not significant at a .10 level. Data analysis will be completed with SPSS 25.0.

We will analyse the qualitative data using thematic analysis [72, 73]. All responses will be transcribed prior to analysis. Themes will be identified using three criteria: recurrence, repetition, and forcefulness [74]. All analyses will be completed by at least two researchers—at least one of whom will be Māori. Specifically, the process will include reading of responses and identifying initial codes. Themes will have to be agreed upon by both researchers for inclusion. Discussion among the coders will be used to clarify and develop themes. The final themes will be reviewed by the research team and advisory boards and shared with participants for validation. Themes will also be compared across the three time periods to explore potential patterns.

#### Intervention evaluation

The purpose of the analysis is to determine whether the peer-educator model results in changes to hauora and mana motukahe between pre-test and post-test. The analysis will compare the two groups and identify if there are intervening variables that explain differential patterns in the changes in the dependent variables*.* The dependent variables are mana motuhake and haurora. The independent variables are the time receiving the orientation programme (T1/T2) and the three time periods (pre-intervention, post T1-intervention, post T2-intervention). Potential intervening variables that will be controlled for include characteristics about the conversations, characteristics of the tuakana/teina, and whether participants talked with each other during the intervention.

The quantitative data are at three levels—repeated measures of variables (three-time periods), individual characteristics of the teina/tuakana, and characteristics about the conversations with the tuakana. The inclusion of multiple levels requires the use of multilevel statistical models to account for clustering. The analysis will account for the nature of the change in the variables along with characteristics about the teina and tuakana that may influence those changes.

Further, we will determine estimates of cost effectiveness using incremental cost effectiveness analysis (ICEA) [75, 76]. Cost-effectiveness analysis involves estimating the cost per unit improvement in the primary outcome variable. We will use HRQOL as an outcome measure for the ICEA [77]. ICEA involves estimating the ratio of the increase in the outcome variable (as measured by the estimated effect size from the intervention trial) to the estimated average cost per participant of the programme (the incremental cost-effectiveness ratio, or ICER). This calculation assumes that the cost of the status quo (no intervention) is zero. ICER is a common measure used in the evaluation of cost-effectiveness of health interventions [78, 79]. The ICER can then be compared with an explicit target (or costs of alternatives) to determine cost effectiveness.

## Discussion

This research project describes the development and research evaluation of an innovative peer-educator model to assist kaumātua addressing life transitions in older age. Underpinning the current project is the will to recognise this potential—the mana motuhake of kaumātua—which directly addresses a desire to value older people in all settings. As the project is co-directed by a Māori community organisation, mana motuhake will be in the hands of kaumātua themselves. The tuakana-teina, peer-education model introduces a Kaupapa Māori approach to social integration and engagement and the strengths-based approach will de-emphasise the disability of kaumātua and centralise kaumātua mana motuhake—their potential, capacity, and ability.

Prospectively, our project presents a transformative approach to reducing the stark inequities in health and wellness outcomes that still exist for kaumātua. Like all people, as kaumātua age they face significant transition points, such as loss of independent living, loss of a spouse, and changing health conditions [1, 9]. Successfully navigating these transitions relies on being able to manage emotional and socio-economic factors, as well as service systems, whilst often being reliant on whānau [10–12]. A disproportionate burden of ageing falls on Māori communities, and without successful navigation, kaumātua are exposed to many negative costs including poor quality of life, social isolation, and poor health outcomes [15, 16]. Transition issues of kaumātua are supported by social, health care, and Māori providers, but many organisations do not have the resources to meet kaumātua needs due to inadequate staffing and high staff turnover. Also, kaumātua desire mana motuhake [20] and often resist support and information provided by someone much younger than themselves. In alignment with mana motuhake, the project looks to Māori culture itself for answers to these disparities. The proposed research’s strengths-based approach highlights the potential of kaumātua mana motuhake by focusing on and valuing Māori epistemologies surrounding ageing [17, 20].

With the ultimate goal of promoting a meaningful life, this peer-education approach essentially encourages self-efficacy for both tuakana and teina, whilst stimulating social integration and engagement. More practically, both parties not only are able to socially interact with each other, the teina receives valued support that may prevent social isolation as kaumātua work through life-events, whilst the tuakana is given further meaning and engagement via the self-efficacy that supporting another can enable. Inherently, the peer-education programme develops age-friendly social environments that value knowledge and cultural concepts such as whakawhanaungatanga (making social connections), tautoko (support), aroha (love) and mātauranga (knowledge) [6, 21].

The mātauranga that emanates from the proposed research will be highly valued by end-users such as other social and health service providers, elderly health researchers, kaumātua groups, iwi, and hapū. As a consequence, the proposed research is also intended to contribute to the development of a new Government strategy (as supported through the Ageing Well National Science Challenge) and broader organisational and community approaches to support the health and social wellbeing of kaumātua. The findings will provide an evidence base for the importance and relevancy of kaumātua mātauranga to create contextually-based and culturally-safe, age-friendly environments that facilitate engagement and participation by kaumātua. If the model is effective, we will seek to facilitate the dissemination and scalability of the intervention. Tuakana-teina is a prominent pedagogical tool stemming from a well-known Māori cultural concept that we believe is scalable not only to other kaumātua communities, but also to Aotearoa’s general population via the notion of ‘peer-education’ grounded in valuing the ageing population and focused on responsibility. Thus, during the last few months of the project we will share the findings widely and establish a value proposition that identifies the beneficial features of the orientation programme, including estimates of cost effectiveness. This community-led dissemination strategy will also involve identifying key people and organisations in the local communities that are inclusive of connections to Māori communities.

### List of Māori words

**Table:** 

Aotearoa	New Zealand
Aroha	Love, compassion, empathy
Hauora	Health
Hapū	Subtribe
Hinengaro	Mind, thought, intellect
Iwi	Tribes
Kaumātua	Elders
Kaupapa Māori	Research by Māori for Māori
Koha	Offering
Kuia	Female elder
Mana	Status and prestige
Mana motuhake	Autonomy, identity and self-actualisation
Māori	Indigenous people of New Zealand
Mātāpono	Principles
Mātauranga	Indigenous knowledge
Noa	Ordinary
Pono	Truth, integrity
Tapu	Sacred
Tautoko	Advocacy, support
Te Ao Māori	Māori worldview
Te Kupenga	Māori Social Survey
Teina	Peer education recipient
Tika	Correctness, justice, fairness
Tikanga	Customs and protocols
Tinana	Body
Tino rangatiratanga	Independence and autonomy
Tuakana	Peer educator
Tuakana-teina	Older sibling/younger sibling
Tuarua	2nd group
Tuatahi	1st group
Wairua	Spirit
Whakawhanaungatanga	Making social connections
Whānau	Extended family
Whanaungatanga	Social health
Whare tapa wha	Four walls of a meeting house

## Additional files

Additional file 1: Questions created for this study for both Tuakana and Teina Questionnaires--organised by construct with English and Māori versions (DOCX 23 kb)

Additional file 2: Questions created for this study for Tuakana Questionnaires only--organised by construct with English and Māori versions (DOCX 16 kb)

## Abbreviations

CBPR: Community-based participatory research
CFA: Confirmatory factor analysis
CFI: Comparative fit index
HRQOL: Health-related quality of life
ICEA: Incremental cost effectiveness analysis
ICER: Incremental cost-effectiveness ratio
RKCT: Rauawaawa Kaumātua Charitable Trust
SRMR: Standardized root mean square residual
T1: 1st group
T2: 2nd group
TLI: Tucker-Lewis index
χ^2^/DF: Chi-square to degrees of freedom

## References

[1] Hayman KJ, Kerse N, Dyall L, Kepa M, Teh R, Wham C (2012). Life and living in advanced age: A cohort study in New Zealand -Te Puawaitanga o Nga Tapuwae Kia Ora Tonu. LiLACS NZ: Study protocol BMC Geriatr.

[2] Blakely T, Shilpi A, Bridget R, Martin T, Martin B (2004). Decades of disparity: widening ethnic mortality gaps from 1980 to 1999. New Zeal Med J.

[3] Howden-Chapman P, Blakely T, Blaiklock AJ, Kiro C (2000). Closing the health gap. New Zeal Med J..

[4] Ministry of Health and University of Otago (2006). Decades of disparity III: ethnic and socioeconomic inequalities in mortality, New Zealand 1981–1999.

[5] Durie MH (1999). Kaumatuatanga: reciprocity: Māori elderly and whanau. New Zeal J Psychol.

[6] Mead H (2003). Tikanga Māori: living by Māori values.

[7] Pihama L, Reynolds P, Smith C, Reid J, Smith L, Tenana R (2014). Positioning historical trauma theory within Aotearoa New Zealand. AlterNative.

[8] Rohr M, Lang F (2009). Aging well together: a mini-review. Gerontology.

[9] Kendig H, Browning C, Thomas S, Wells Y (2014). Health, lifestyle, and gender influences on aging well: an Australian longitudinal analysis to guide health promotion. Front Public Health.

[10] Fowler C, Gasiorek J, Giles H (2015). The role of communication in aging well: introducing the communicative ecology model of successful aging. Commun Monogr.

[11] Oetzel JG, Simpson M, Berryman K, Iti T, Reddy R (2015). Managing communication tensions and challenges during the end-of-life journey: perspectives of Māori kaumātua and their whānau. Health Commun.

[12] Oetzel JG, Simpson M, Berryman K, Reddy R (2015). Differences in ideal communication behaviours during end-of-life care for Māori carers/patients and palliative care workers. Palliat Med.

[13] Maclennan B, Wyeth E, Hokowhitu B, Wilson S. Derrett S. Injury severity and 3-month outcomes among Māori: results from a New Zealand prospective cohort study. New Zeal Med J. 2013; 126(1379):39–49. 24045351

[14] Wyeth E, Derrett S, Hokowhitu B. Samaranayaka A. Indigenous injury outcomes: life satisfaction among injured Māori in New Zealand three months after injury. Health Qual Life Outcomes 2013; 11:120–135. 10.1186/1477-7525-11-120 PMC3720216 23866834

[15] Wham C, Teh R, Moyes S, Dyall L, Kēpa M, Hayman K, Kerse N (2015). Health and social factors associated with nutrition risk: results from life and living in advanced age: a cohort study in New Zealand (LiLACS NZ). J Nutr Health Aging.

[16] Dyall L, Kepa M, Teh R, Mules R, Moyes SA, Wham C (2014). Cultural and social factors and quality of life of Maori in advanced age. Te puawaitanga o nga tapuwae kia ora tonu - life and living in advanced age: a cohort study in New Zealand (LiLACS NZ). New Zeal Med J.

[17] Hokowhitu B, Kermoal N, Andersen C, Reilly M, Rewi P, Petersen A (2010). Indigenous identity and resistance: researching the diversity of knowledge.

[18] Hokowhitu B, Ka’ai T, Moorfield J, Reilly M (2003). Te tāminga o te mātauranga Māori: colonisation in education. Ki te whaiao: an introduction to Māori society.

[19] Taskforce of Whānau-centred initiatives. Whanau ora: Report of the taskforce on Whanau-centred initiatives. Wellington: Ministry of Health; 2010.

[20] Hokowhitu B (2009). Indigenous existentialism and the body. Cult Stud Rev.

[21] Winitana M (2012). Remembering the deeds of Māui: what messages are in the tuakana-teina pedagogy for tertiary educators?. MAI Review.

[22] Tate H (2010). Towards some foundations of a systematic Māori theology: he tirohanga anganui ki ētahi kaupapa hōhonu mō te whakapono Māori.

[23] Bandura A (1986). Social foundations of thought and action: a social cognitive theory.

[24] Ajzen I, Fishbein M (1980). Understanding attitudes and predicting social behavior.

[25] Rogers E (2003). Diffusion of innovations.

[26] Braun KL, Kagawa-Singer M, Holden AEC, Burhansstipanov L (2012). Cancer patient navigator tasks across the cancer care continuum. J Health Care Poor Underserved.

[27] Oman RF, Vesely S, Aspy CB, McLeroy KR, Rodine S, Marshall L (2004). The potential protective effects of youth assets on adolescents' alcohol and drug use. Am J Public Health.

[28] Gottfredson DC, Wilson DB (2003). Characteristics of effective school-based substance abuse prevention. Prev Sci.

[29] Karwalajtys T, McDonough B, Hall H, Guirguis-Younger M, Chambers LW, Kaczorowski J (2009). Development of the volunteer peer educator role in a community cardiovascular health awareness program (CHAP): a process evaluation in two communities. J Community Health.

[30] Dennis C-L (2003). Peer support within a health care context: a concept analysis. Int J Nurs Stud.

[31] Philis-Tsimikas A, Fortmann A, Lleva-Ocana L, Walker C, Gallo LC (2011). Peer-led diabetes education programs in high-risk Mexican Americans. Diabetes Care.

[32] Layne JE, Sampson SE, Mallio CJ, Hibberd PL, Griffith JL, Krupa DS (2008). Successful dissemination of a community-based strength training program for older adults by peer and professional leaders: the people exercising program. J Am Geriatr Soc.

[33] Uitewaal P, Bruijnzeels M, de Hoop T, Hoes A, Thomas S (2004). Feasibility of diabetes peer education for Turkish type 2 diabetes patients in Dutch general practice. Patient Educ Couns.

[34] Khong L, Farringdon F, Hill K, Hill A (2015). "we are all one together": peer educators' views about falls prevention education for community-dwelling older adults - a qualitative study. BMC Geriatr.

[35] Little G (2012). Nordic walking for health in wales: an innovative and successful active ageing programme for older people. J Aging Phys Act.

[36] Stevens Z, Barlow C, Lliffe S (2015). Promoting physical activity among older people in primary care using peer mentors. Prim Health Care Res Dev.

[37] Werner D, Teufel J, Brown SL (2014). Evaluation of a peer-led, low-intensity physical activity program for older adults. Am J Health Educ.

[38] Kocken P, Voorham A (1998). Effects of a peer-led senior health education program. Patient Educ Couns.

[39] Goodman C, Iliffe S, Manthorpe J, Gage H, Barclay S, Mathie E (2011). Talking about living and dying with the oldest old: public involvement in a study on end of life care in care homes. BMC Palliat Care.

[40] Lorig K, Hurwicz M, Sobel D, Hobbs M, Ritter P (2005). A national dissemination of an evidence-based self-management program: a process evaluation study. Patient Educ Couns.

[41] Seymour JE, Almack K, Kennedy S, Froggatt K (2013). Peer education for advance care planning: volunteers’ perspectives on training and community engagement activities. Health Expect.

[42] Uitewaal P, Manna D, Bruijnzeels M, Hoes A, Thomas S (2004). Prevalence of type 2 diabetes mellitus, other cardiovascular risk factors, and cardiovascular disease in Turkish and Moroccan immigrants in North West Europe: a systematic review. Prev Med.

[43] Robinson E, Rankin S, Arnstein P, Carroll D, Traynor K (1998). Meeting the needs of unpartnered elders: a peer training program involving elders with myocardial infarction. Prog Cardiovasc Nurs.

[44] Schwartz C, Sendor R (1999). Helping others helps oneself: response shift effects in peer support. Soc Sci Med.

[45] Tuhiwai-Smith L (1999). Decolonizing methodologies: research and indigenous peoples.

[46] Smith GH (1997). The development of kaupapa Māori: theory and praxis.

[47] Wallerstein N, Duran B, Oetzel JG, Minkler M (2018). Community-based participatory research for health: advancing social and health equity, 3rd edition.

[48] Oetzel JG, Villegas M, Zenone H, White Ha E, Wallerstein N, Duran B (2015). Enhancing stewardship of community-engaged research through governance. Am J Public Health.

[49] Wyeth E, Derrett S, Hokowhitu B, Langely J, Hall C (2010). Rangatiratanga and oritetanga: responses to the treaty of Waitangi in a New Zealand study. Ethn Health.

[50] Kennedy V, Cram F (2010). Ethics of researching with whānau collectives. MAI Review..

[51] Mane J (2009). Kaupapa Māori: a community approach. MAI Review..

[52] Durie M (2005). Ngā tai matatū: tides of Māori endurance.

[53] Walker H. Kai te ao marama. Kai ro pouri tonu ranei: how enlightened are we? Wellington. New Zealand: Victoria University; n.d.

[54] Sanders C, Seymour J, Clarke A, Gott M, Welton M (2006). Development of a peer education programme for advance end-of-life care planning. International J Palliat Nurs.

[55] Clarke A, Sanders C, Seymour J, Gott M, Welton M (2009). Evaluating a peer education programme for advance end-of-life care planning for older adults: the peer educators' perspective. Int J Disabil Hum Dev.

[56] Patsopoulos NA (2011). A pragmatic view on pragmatic trials. Dialogues Clin Neurosc.

[57] Snijder T, Bosker RJ (1999). Multilevel analysis: an introduction to basic and advanced multilevel modeling.

[58] Raudenbush S, Liu X, Congdon R, Spybrook J (2004). Optimal design for longitudinal and multilevel research: documentation for the optimal design software.

[59] Rochford T (2004). Whare tapa wha: a Māori model of a unified theory of health. J Prim Prev.

[60] Achat HM, Thomas P, Close GR, Moerkerken LR, Harris MF (2010). General health care service utilisation: where, when and by whom in a socioeconomically disadvantaged population. Aust J Prim Health.

[61] Dulin PL, Stephens C, Alpass F, Hill RD, Stevenson B (2011). The impact of socio-contextual, physical and lifestyle variables on measures of physical and psychological wellbeing among Māori and non-Māori: the New Zealand health. Work and Retirement Study Ageing Soc.

[62] Wu A, Revicki D, Jacobsen D, Malitz F (1997). Evidence for reliability, validity and usefulness of the medical outcomes study HIV health survey (MOS-HIV). Qual Life Res.

[63] Aotearoa T (2013). Te Kupenga 2013: A survey of Māori well-being.

[64] Unger J, McAvay G, Bruce ML (1999). Variation in the impact of social network characteristics on physical functioning in elderly persons: MacArthur studies of successful aging. J Gerontol B Psychol Sci Soc Sci.

[65] Oeki M, Mogami T, Hagino H (2012). Self-perceived burden in patients with cancer: scale development and descriptive study. Eur J Oncol Nurs.

[66] Kroemeke A (2015). Perceived autonomy in old age scale: factor structure and psychometric properties of the polish adaptation. Psychiatr Pol.

[67] Cantril H (1965). The pattern of human concerns.

[68] Windsor T, Curtis R, Luszcz MA (2015). Sense of purpose as a psychological resource for aging well. Dev Psychol.

[69] Bandura A, Urdan PT (2006). Guide for constructing self-efficacy scales. Self-efficacy beliefs of adolescents.

[70] Sahinidis A, Bouris J (2008). Employee perceived training effectiveness relationship to employee attitudes. J Eur Industrial Train.

[71] Kline R (2011). Principles and practice of structural equation modeling.

[72] Braun V, Clarke V (2006). Using thematic analysis in psychology. Qual Res Psychol.

[73] Patton MQ (2002). Qualitative research and evaluation methods.

[74] Owen W (1984). Interpretive themes in relational communication. Q J Speech.

[75] Gold MR, Siegel JE, Russell LB, Weinstein MC (1996). Cost-effectiveness in health and medicine.

[76] Edejer T, Baltussen R, Adam T, Hutubessy R, Acharya A, Evans D (2003). Making choices in health: WHO guide to cost-effectiveness analysis.

[77] Sindelar J, Jofre-Bonet M, French M, McLellan A (2004). Cost effectiveness analysis of addiction treatment: paradoxes of multiple outcomes. Drug Alcohol Depen.

[78] Medley A, Kennedy C, O'Reilly K, Sweat M (2009). Effectiveness of peer education interventions for HIV prevention in developing countries: a systematic review and meta-analysis. AIDS Educ Prev.

[79] Melis R, Adang E, Teerenstra S, van Eijken M, Wimo A, van Achterberg T (2008). Cost-effectiveness of a multidisciplinary intervention model for community-dwelling frail older people. J Gerontol A Biol Sci Med Sci.

