# Supplementary material for: Kaumātua Mana Motuhake: A study protocol for a peer education intervention to help Māori elders work through later-stage life transitions
Source: BMC Geriatr. 2019 Feb 7;19:36. doi: 10.1186/s12877-019-1041-2 (PMC6367813; doi:10.1186/s12877-019-1041-2)
Supplement: Supplementary file 1 — Questions created for this study for both Tuakana and Teina Questionnaires--organised by construct with English and Māori versions (DOCX 23 kb) [file 12877_2019_1041_MOESM1_ESM.docx]

**Questions Created for this Study: Constructs in both Tuakana and Teina Questionnaires**

**Knowledge of Health and Social Services**

1. Do you know where to go to get services for your housing, health, social needs?/ Tēnā, e mōhio pai ana koe me haere ki whea kimi āwhina ai mō tō whare, mō tō hauora, mō ō hiahiatanga rānei?

- Yes- Āe
- No- Kāo
- Sometimes- I ētahi wā

If yes, where would you go?/ Mehemea āe me haere koe ki whea

1. Would you like/prefer someone to go with and support you when you seek services?/ Ki te haere koe ki te kimi āwhina he pai ki a koe mehemea ka haere mai (t)ētahi atu tāngata hoki ki te āwhina i a koe?

- Yes- Āe
- No- Kāo
- Sometimes- I ētahi wā

1. How likely are you to get help with a housing, health, and social need if you need it?/ Tēnā, ki te hiahia āwhina mō tō whare, mō tō hauora, mō ō hiahiatanga rānei, ka tutuki i a koe tēnā āwhina mehemea ka hiahia?

| Extremely likely/ Āe, tino tutuki | Very likely/ Āe,  ka tutuki | Somewhat likely/ Ka āhua tutuki | Not very likely/ Kāore pea e tutuki | Not at all/ Kāore i paku tutuki |
| --- | --- | --- | --- | --- |

**Loneliness**

1. How often do you feel connected to others?/ Ka pēwhea te kaha o tō honohonotanga/whanaungatanga ki ētahi atu tāngata?

| Always feel connected/ Ka honohono au i ngā wā katoa | Mostly feel connected/ Ka honohono au i te nuinga o te wā | Sometimes feel connected/ Ka honohono au i ētahi wā | Never feel connected/ Kāore au i paku honohono |
| --- | --- | --- | --- |

**Perceived Benefit**

1. My whānau accepts the knowledge I share with them./ Ka taea e au te tohatoha/ te hoatu ōku mātauranga ki tōku whānau.

| Always/ I ngā wā katoa | Most of the time/ I te nuinga o te wā | Sometimes/ I ētahi wā | Never/ Karekau |
| --- | --- | --- | --- |

1. I feel my whānau benefits from having me around./ Ki a au nei he hua tōku oranga mō tōku whānau.

| Always/ I ngā wā katoa | Most of the time/ I te nuinga o te wā | Sometimes/ I ētahi wā | Never/ Karekau |
| --- | --- | --- | --- |

**Economic Wellbeing**

1. Does someone else have financial control of your resources because they have a bank card to use, they share an account with you, or because they have power of attorney?/ Kei (t)ētahi tāngata te mana whakahaere o āu rauemi/pūtea, nā te mea, kei a ia te kāri pūtea, ka tohatoha pūtea kōrua, kei a ia te mana whakahaere pūtea rānei ?

- Yes- Āe
- No- Kāo

If yes, has this created any problems for you?/ Mehemea ka kī mai āe, kua pā mai ētahi raruraru ki a koe?

- Yes- Āe
- No- Kāo

**Satisfaction with Interaction**

Think about your interaction with your teina/tuakana. How satisfied are you with the following? / Me whakaaro ake e koe ki tō noho tahi ki tō teina. Ka pēhea te nui o tō manawareka ki ngā mea e whai ane nei?

1. The conversations that you had/ Ngā kōrerorerotanga

| Extremely satisfied/ Tino eke ōku manawareka | Satisfied/ I eke ōku manawareka | Somewhat satisfied/ I āhua eke ōku manawareka | Dissatisfied/ Kāore i eke ōku manawareka | Extremely dissatisfied/ Kāore i tino eke ōku manawareka |
| --- | --- | --- | --- | --- |

1. The amount of time spent/ Te whakapaunga o te wā

| Extremely satisfied/ Tino eke ōku manawareka | Satisfied/ I eke ōku manawareka | Somewhat satisfied/ I āhua eke ōku manawareka | Dissatisfied/ Kāore i eke ōku manawareka | Extremely dissatisfied/ Kāore i tino eke ōku manawareka |
| --- | --- | --- | --- | --- |

1. The advice or recommendations provided/ Te āhua o ngā kōrero kua whārikihia

| Extremely satisfied/ Tino eke ōku manawareka | Satisfied/ I eke ōku manawareka | Somewhat satisfied/ I āhua eke ōku manawareka | Dissatisfied/ Kāore i eke ōku manawareka | Extremely dissatisfied/ Kāore i tino eke ōku manawareka |
| --- | --- | --- | --- | --- |

**Open-ended Questions**

1. What are the biggest concerns that you currently have as you grow older?/ Tēnā, he aha ō āwangawanga nui, i a koe e kaumatua haere ana?
2. What are the concerns that you see developing in the next 3-5 years?/ Ki ō whakaaro he aha ngā āwangawanga nui ka ara ake pea i roto i ngā tau e toru, e rima kei te heke mai?
3. What are the best things about your life right now?/ He aha ngā mea tino papai rawa atu o tō oranga ināianei
4. What would help you improve your life?/ He aha ngā mea hei whakapiki ake i tō oranga?
5. Mana motuhake is about being in control of your own life including spiritual, emotional, physical, and relational elements. How would you describe your mana motuhake?/ Ko te mana motuhake e tohu ana i te mana whakahaere o tō oranga, pērā i te taha wairua, te taha tinana, te taha hinengaro. Ka pēwhea te kaha o tō mana motuhaketanga? Tēnā, whakamārama mai.

**Demographics**

1. What is your relational status?/ Ko tēwhea o ēnei e tika ana mōu?

- Married-Kua marena
- Widower/widowed- Kua mate tōku hoa rangatira
- Divorced/separated- Kua wehe māua
- Single- Kāore ōku hoa rangatira

1. Are you looking after your mokopuna or other children?/ Kei te manaaki/tiaki koe i āu mokopuna, i ētahi atu tamariki rānei?

- Yes- Āe
- No- Kāo
- Sometimes- I ētahi wā

If yes or sometimes, are you the primary caregiver for them?/ Mehemea āe ranei 󠆛I ētahi wā, ko koe te kaitiaki matua o rātou?

- Yes- Āe
- No- Kāo

1. Who usually lives in your house with you? Check all that apply/ Ko wai ngā tāngata e noho ana ki tō taha, ki roto i tō whare hoki? Whiriwhiria ngā mea e tika ana.
   - Partner- Ko tōku hoa rangatira
   - Children- Ko aku tamariki
   - Mokopuna- Ko aku mokopuna
   - Flatmate- Ko ōku hoa noho
   - Other whanau- Ko ētahi atu whānau
