# Supplementary material for: Kaumātua Mana Motuhake: A study protocol for a peer education intervention to help Māori elders work through later-stage life transitions
Source: BMC Geriatr. 2019 Feb 7;19:36. doi: 10.1186/s12877-019-1041-2 (PMC6367813; doi:10.1186/s12877-019-1041-2)
Supplement: Supplementary file 2 — Questions created for this study for Tuakana Questionnaires only--organised by construct with English and Māori versions (DOCX 16 kb) [file 12877_2019_1041_MOESM2_ESM.docx]

**Questions Created for this Study: Constructs for Tuakana Questionnaire Only**

**Self-efficacy**

1. I can help other kaumatua who are dealing with a loss of spouse./ Ka taea e au te āwhina atu i (t)ētahi kaumatua mehemea kua mate tōna hoa rangatira.

| Cannot do at all/ Kāore e taea e au | Not sure if I can do it/ Kāore au e mōhio mehemea ka taea e au | Moderately certain I can do it/Tērā pea, ka taea e a | Mostly certain I can do it/ Ka taea e au | Completely certain I can do it/ Tino whakapono ka taea e au |
| --- | --- | --- | --- | --- |

1. I can help other kaumatua who are entering retirement./ Ka taea e au te āwhina i ngā kaumātua e rītaia haere ana.

| Cannot do at all/ Kāore e taea e au | Not sure if I can do it/ Kāore au e mōhio mehemea ka taea e au | Moderately certain I can do it/Tērā pea, ka taea e a | Mostly certain I can do it/ Ka taea e au | Completely certain I can do it/ Tino whakapono ka taea e au |
| --- | --- | --- | --- | --- |

1. I can help other kaumatua who are dealing with the loss of their driver’s licence./ Ka taea e au te āwhina i ngā kaumātua kua ngaro ā rātou raihana taraiwa waka.

| Cannot do at all/ Kāore e taea e au | Not sure if I can do it/ Kāore au e mōhio mehemea ka taea e au | Moderately certain I can do it/Tērā pea, ka taea e a | Mostly certain I can do it/ Ka taea e au | Completely certain I can do it/ Tino whakapono ka taea e au |
| --- | --- | --- | --- | --- |

1. I can help other kaumatua who are facing a significant change in health./ Ka taea e au te āwhina i ngā kaumātua kua tino heke ō rātou oranga/hauora.

| Cannot do at all/ Kāore e taea e au | Not sure if I can do it/ Kāore au e mōhio mehemea ka taea e au | Moderately certain I can do it/Tērā pea, ka taea e a | Mostly certain I can do it/ Ka taea e au | Completely certain I can do it/ Tino whakapono ka taea e au |
| --- | --- | --- | --- | --- |

1. I can help other kaumatua who are facing moving away from independent living./ Ka taea e au te āwhina i ngā kaumātua kua ngaro ō rātou motuhaketanga, ka mutu, kua nuku ki whare/wāhi kē noho ai.

| Cannot do at all/ Kāore e taea e au | Not sure if I can do it/ Kāore au e mōhio mehemea ka taea e au | Moderately certain I can do it/Tērā pea, ka taea e a | Mostly certain I can do it/ Ka taea e au | Completely certain I can do it/ Tino whakapono ka taea e au |
| --- | --- | --- | --- | --- |

**Perceived Effectiveness of Orientation Programme**

1. How effective do you feel the orientation programme was?/ Ki ō whakaaro i tutuki pai te mahi whakangungu?

| Very effective. Āe, i tutuki rawa atu | Effective/ Āe, i tutuki pai | Neither effective or not effective/ Kāore i aha | Ineffective/ Kāore i tutuki | Very ineffective/ Kāore i paku tutuki |
| --- | --- | --- | --- | --- |

1. Did you consider this to be time spent well?/ Ki ō whakaaro i whai hua mai i tēnei mahi whakangungu?
2. How relevant was this training for helping kaumatua?/ Ki ō whakaaro i tino hāngai tēnei mahi whakangungu kia tautoko mārika i ngā kāumatua?
3. Do you have a desire for additional training?/ Kei te hiahia anō ētahi atu mahi whakangungu?

| Yes need more training to be effective/ Āe, me whakawhānui ake te mahi whakangungu kia eke ai taku mahi | Yes some more would be nice in the future, but I’m ready to be a tuakana now/ Āe, engari ā tōna wā, kua rite au ināianei kia tū hei tuakana | No I’m fine/ Kāo, e pai ana ahau |
| --- | --- | --- |
